# CARMA3 Is a Critical Mediator of G Protein-Coupled Receptor and Receptor Tyrosine Kinase-Driven Solid Tumor Pathogenesis

**DOI:** 10.3389/fimmu.2018.01887

**Published:** 2018-08-15

**Authors:** J. Randall McAuley, Tanner J. Freeman, Prasanna Ekambaram, Peter C. Lucas, Linda M. McAllister-Lucas

**Affiliations:** ^1^Department of Pediatrics, Division of Pediatric Hematology-Oncology, University of Pittsburgh School of Medicine, Pittsburgh, PA, United States; ^2^Department of Pathology, University of Pittsburgh School of Medicine, Pittsburgh, PA, United States

**Keywords:** CARMA3, Bcl10, MALT1, G protein-coupled receptor, receptor tyrosine kinases, NF-κB, cancer

## Abstract

The CARMA–Bcl10–MALT1 (CBM) signalosome is an intracellular protein complex composed of a CARMA scaffolding protein, the Bcl10 linker protein, and the MALT1 protease. This complex was first recognized because the genes encoding its components are targeted by mutation and chromosomal translocation in lymphoid malignancy. We now know that the CBM signalosome plays a critical role in normal lymphocyte function by mediating antigen receptor-dependent activation of the pro-inflammatory, pro-survival NF-κB transcription factor, and that deregulation of this signaling complex promotes B-cell lymphomagenesis. More recently, we and others have demonstrated that a CBM signalosome also operates in cells outside of the immune system, including in several solid tumors. While CARMA1 (also referred to as CARD11) is expressed primarily within lymphoid tissues, the related scaffolding protein, CARMA3 (CARD10), is more widely expressed and participates in a CARMA3-containing CBM complex in a variety of cell types. The CARMA3-containing CBM complex operates downstream of specific G protein-coupled receptors (GPCRs) and/or growth factor receptor tyrosine kinases (RTKs). Since inappropriate expression and activation of GPCRs and/or RTKs underlies the pathogenesis of several solid tumors, there is now great interest in elucidating the contribution of CARMA3-mediated cellular signaling in these malignancies. Here, we summarize the key discoveries leading to our current understanding of the role of CARMA3 in solid tumor biology and highlight the current gaps in our knowledge.

## Introduction

**Figure 1:**
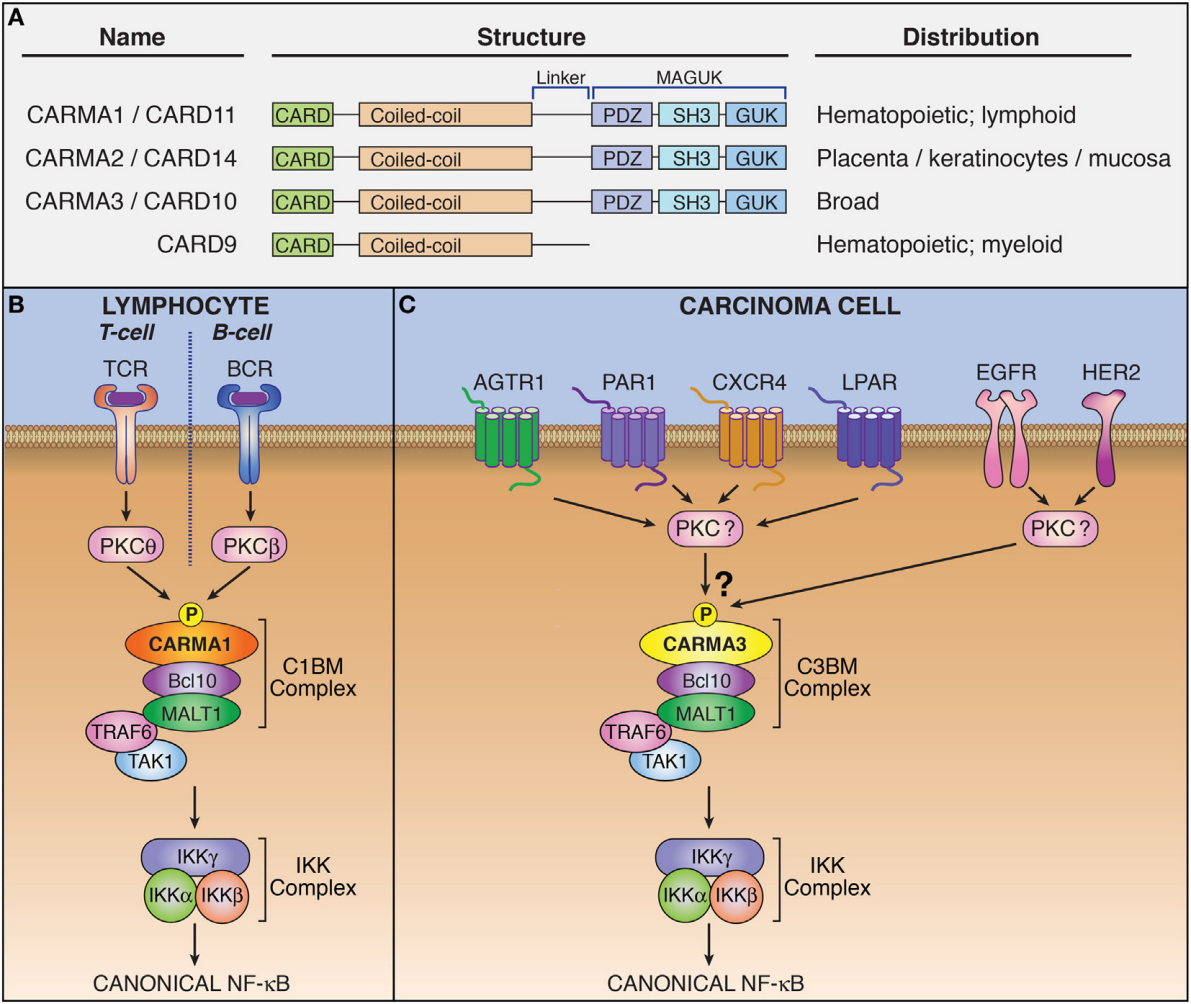
The CARMA3-containing CARMA–Bcl10–MALT1 (CBM) complex mediates signaling downstream of specific G protein-coupled receptors (GPCRs) and receptor tyrosine kinases (RTKs). **(A)** Domain structure and tissue distribution of CARMA protein family members. **(B)** Schematic of lymphocyte antigen receptor-dependent CARMA1-containing CBM complex signaling. **(C)** Schematic of CARMA3-containing CBM complex signaling.

In the late 1990s, a large family of proteins containing a homophilic protein–protein interaction module referred to as a “caspase activation and recruitment domain” (CARD) was discovered (1). While initially associated with apoptotic signaling, several proteins in this family were eventually linked to activation of the NF-κB transcription factor. In 2000, CARD9 was identified as a protein that directly interacts with the CARD of Bcl10, a signaling protein involved in regulating NF-κB (2). Subsequently in 2001, three proteins structurally related to CARD9, namely CARMA3 (also referred to as CARD10 and Bimp1), CARMA1 (CARD11 and Bimp3), and CARMA2 (CARD14 and Bimp2), was discovered as a unique family of proteins that contained both CARD domains and membrane-associated guanylate kinase domains (Figure 1A) (3–6). Each of these proteins was shown to engage in CARD–CARD interaction with Bcl10 to enact canonical, IKK complex-associated NF-κB activation. The notion of a tripartite CARMA-containing signaling complex came with the discovery that a third protein, MALT1, interacted with Bcl10 to activate NF-κB (7–9). This complex is now referred to as the CARMA–Bcl10–MALT1 (CBM) signalosome.

CARMA family members appear to be functionally similar, albeit with differing expression patterns (5, 10). CARMA1 is found primarily in hematopoetic cells. CARMA2 is present in placenta, skin, and several mucosal tissues including buccal mucosa, esophagus, and pharyngeal mucosa, while CARMA3 is widely expressed in many tissues but not in hematopoietic cells (11). The upstream signaling events that regulate formation of the CBM complex were originally elucidated in lymphocytes for the CARMA1-containing CBM. Specifically, lymphocyte antigen receptor stimulation leads to activation of protein kinase C (PKC), which subsequently phosphorylates CARMA1 (12, 13). This phosphorylation triggers a conformational change in CARMA1 that allows it to form a polymeric complex with Bcl10/MALT1 (Figure 1B). Different PKC isoforms phosphorylate CARMA1 in different cell subtypes: PKCβ in B cells and PKCθ in T cells (12, 13). While specific PKC-mediated signaling events upstream of the CBM complex have not been elucidated for CARMA2 and CARMA3-containing complexes, PKC agonists such as phorbol myristate acetate and ionomycin (14) have been shown to activate CBM signaling in CARMA3-containing cells, suggesting that this phosphorylation-dependent mechanism for activation of the CARMA3–CBM complex is likely similar to that of the CARMA1–CBM complex (15).

CARMA1 phosphorylation triggers CBM complex oligomerization (16–21). Phosphorylated CARMA1 nucleates a very long filament of Bcl10 which is decorated around the outside of the filament by MALT1. MALT1 serves as the effector protein of the CBM complex and possesses at least two distinct functions by which it carries out downstream signaling: First, MALT1 functions as a scaffold, capable of engaging in protein–protein interactions and second, MALT1 functions as a protease. As a scaffold, activated MALT1 interacts with components of the NF-κB signaling machinery including the TRAF6 ubiquitin ligase (22–24). These interactions result in the polyubiquitination of IKKγ and the subsequent activation of the IKK complex (25, 26). The active IKK complex then induces the phosphorylation of Inhibitor of κB (IκB), targeting IκB for proteasomal degradation and freeing NF-κB transcription factor dimers to translocate into the nucleus and modulate the transcription of target genes (9). As a protease, MALT1 cleaves multiple specific substrates, including the NF-κB family transcription factor subunit RelB, and the deubiquitinases, CYLD and A20 (27–29). [For a detailed description of the multiple known MALT1 substrates, please refer to recent reviews (30, 31).] The effects of MALT1 protease activity are not yet completely understood, and many current studies suggest that one of the consequences of MALT1-dependent substrate cleavage is to maximize and sustain signaling by inactivating inhibitors of the NF-κB pathway. MALT1 proteolytic activity is transient after receptor triggering and the kinetics of MALT1 protease-dependent effects on cellular signaling has not yet been analyzed in detail.

CARMA1, Bcl10, and MALT1 are each *bona fide* proto-oncoproteins, since recurrent chromosomal translocations and gain-of-function mutations that lead to deregulation of these signaling proteins have been identified in B-cell lymphoma. Three specific recurrent chromosomal translocations were discovered in the B-cell malignancy, MALT lymphoma, and each involves components of the CBM complex: the first described was the t(11;18) resulting in expression of the API2–MALT1 fusion oncoprotein (32–34). Two other translocations were also identified, each involving the Ig heavy chain enhancer: t(1;14) and t(14;18), placing the intact Bcl10 and MALT1 genes, respectively, under control of the enhancer and leading to their overexpression (35, 36). In activated B-cell type diffuse large B-cell lymphoma (ABC-DLBCL), activating mutations in B-cell receptor subunits are present in approximately 23% of cases and result in constitutive BCR-driven CBM-dependent NF-κB activity (37–39). Gain-of-function mutations of CARMA1 occur in another 9% of cases and similarly result in constitutive CBM activity. More recent studies have suggested a role for constitutive CBM activity in driving other lymphoid cancers beyond MALT lymphoma and ABC-DLBCL. For example, activating mutations in CARMA1 have been identified in a subset of T-cell malignancies including adult T-cell leukemia/lymphoma, peripheral T cell lymphoma, and Sezary syndrome (40). Also, in the B-cell malignancy, primary effusion lymphoma, the Kaposi sarcoma herpes virus is thought to utilize the K13 viral oncoprotein to form a “KBM” complex composed of K13, Bcl10, and MALT1 to promote lymphomagenesis (41). In each of these malignancies, either viral oncogenes, oncogenic mutations, or chromosomal translocations drive deregulated inappropriate NF-κB activity (9).

With the identification of these CBM-associated genetic aberrations, the vast majority of research on the CBM complex in cancer has been focused on lymphoma. Over the last decade, a large number of studies have demonstrated a critical role for the CARMA3-containing CBM complex in solid tumors. Here, we summarize the key findings that have led to our current understanding of the role of CARMA3 in solid tumor biology.

## A CARMA3-Containing CBM Complex Operates Downstream of G Protein-Coupled Receptors (GPCRs) in Non-Immune Cells

Early investigations showed that CARMA1 expression is predominantly restricted to lymphoid tissue, while CARMA3 is more widely expressed (3–6). Subsequent bioinformatic analysis revealed that CARMA3 is in fact expressed in almost all tissue types, including kidney, heart, brain, liver, and many other tissues of ectodermal, mesodermal, and endodermal origin, as are Bcl10 and MALT1. The only exception appears to be tissues dominated by cells of hematopeoitic origin, such as spleen, thymus, and lymph node, where CARMA1 predominates (10). CARMA1 and CARMA3 share both structural and functional similarity, and CARMA3 is able to complement CARMA1 deficiency in T-cells, rescuing the NF-κB defect (42). Given the similarities between CARMA1 and CARMA3, we and two other groups sought to investigate whether CARMA3 fulfilled a similar functional role to that of CARMA1, but in a different cellular context and downstream of receptors other than the lymphocyte antigen receptor. Indeed, we found that the CARMA3-containing CBM complex (*abbreviated henceforth as C3BM*) can promote NF-κB activation in cells outside of the immune system. These initial studies all demonstrated that the C3BM complex operates downstream of specific GPCRs that are known to stimulate PKC (Figure 1C) (43–45). Our own laboratory’s initial studies revealed that the C3BM complex could be activated by a GPCR that serves as a receptor for the peptide hormone, angiotensin II (AGTR1), while others focused initially on the lysophosphatidic acid receptor (LPAR) family (43, 45), and the receptor for endothelin-1 (EDNRA; also known as ET_A_R) (45). Since the time of these initial discoveries in 2007, the list of GPCRs that signal *via* the C3BM complex has continued to expand to include a wide array of receptors such as chemokine receptor type 2 (CXCR2) (46), CXCR4 (47), platelet-activating factor receptor (PTAFR; also known as PAF-R) (48), and protease-activated receptor 1 (F2R; also known as PAR1) (49) (Figure 1C; Table 1). Because inappropriate GPCR activation underlies the pathogenesis of many inflammatory and malignant disorders, these discoveries have led to notion that the CARMA3-containing CBM signalosome may represent a promising new target for therapeutic inhibition in GPCR-driven inflammation and cancer.

**Table 1:** Receptors that signal *via* CARMA3.

(A) G protein-coupled receptors (GPCRs) in solid tumors			

Receptor	Ligand	Tumor-type	Reference
AGTR1	Angiotensin II	Breast cancer	Ekambaram et al. (50)
CXCR4	CXCL12/SDF-1	Oral squamous cell carcinoma	Rehman and Wang (47)
LPAR	LPA	Ovarian cancer	Mahanivong et al. (51)

**(B) GPCRs in non-malignant cells**			

**Receptor**	**Ligand**	**Disease/cellular system**	**Reference**

AGTR1	Angiotensin II	Vascular inflammation and atherogenesis	McAllister-Lucas et al. (52)
		Hepatocytes	McAllister-Lucas et al. (44)
CXCR2	CXCL8/IL8	Endothelial cells	Martin et al. (46)
ET_A_R	Endothelin-1	Mouse embryonic fibroblasts (MEFs)	Grabiner et al. (53)
LPAR	LPA	Asthma	Medoff et al. (54) and Causton et al. (55)
		MEFs	Grabiner et al. (53)
PAR1	Thrombin	Endothelial cells	Delekta et al. (49) and Klei et al. (56)
PAF-R	PAF	Intestinal epithelial cells	Borthakur et al. (48)

**(C) Receptor tyrosine kinases in solid tumors**			

**Receptor**	**Ligand**	**Tumor-type**	**Reference**

EGFR	EGF	Non-small cell lung carcinoma	Li et al. (57)
		Breast and other cancer cells	Jiang et al. (58)
HER2		Breast cancer	Pan et al. (59)

### C3BM Signaling Plays a Critical Role in GPCR-Driven Solid Tumors

G protein-coupled receptor signaling has long been associated with oncogenesis and cancer progression (60). The discovery that the C3BM complex functions downstream of GPCRs to activate the pro-oncogenic NF-κB transcription factor in non-immune cells raised the possibility that the CARMA3-containing CBM complex might play a role in solid tumor pathogenesis. Indeed, C3BM complex signaling has now been demonstrated to have protumorigenic effects in several tumor types associated with dysregulated GPCR signaling. Furthermore, deficiency of C3BM complex components appear to blunt cancer growth in a range of model systems, indicating a possible therapeutic role for targeting C3BM complex signaling in the treatment of selected solid tumors. Below, we discuss the specific GPCRs currently known to stimulate C3BM signaling in solid tumors.

#### LPAR

Lysophosphatidic acid (LPA) is a lipid signaling molecule that plays an important role in a diverse array of biological contexts ranging from tissue development and remodeling to pathological processes including reactive airway disease and cancer cell proliferation (54, 55, 61–63). Acting in an autocrine or paracrine manner, LPA can engage a family of GPCRs, LPARs 1–6, to stimulate various intracellular signaling pathways, including activation of NF-κB. Synthetic enzymes responsible for generating LPA, such as autotaxin and lipase member 1 (LIPI), are highly upregulated in multiple aggressive tumors including breast cancer, ovarian cancer, and metastatic Ewing’s sarcoma (62, 64). In addition, deregulation of the LPAR–NF-κB signaling pathway has been implicated in driving a variety of tumors, including breast (63), colon (65), and prostate cancers (66, 67).

One of the first indications of a role for the C3BM complex in non-immune cells was the finding that LPA induction of NF-κB in mouse embryonic fibroblast (MEF) cells requires Bcl10 (43, 45). A subsequent study then demonstrated that the C3BM complex plays an important role in the pathophysiology of LPA-induced ovarian cancer cell migration and invasion (Table 1A) (51). These authors showed that siRNA-mediated knockdown of either CARMA3, Bcl10, or MALT1 suppressed LPA-induced NF-κB activation in ovarian cancer cell models. Furthermore, suppression of CBM signaling through the expression of a dominant-negative CARMA3 attenuated LPA-induced ovarian cancer cell invasion. Intriguingly, a dominant-negative variant of PKCα inhibited LPA-induced NF-κB activation in this system, suggesting that this PKC isoform might have a role in upstream regulation of the CARMA3-containing CBM complex in ovarian cancer cells. This study, published in 2008, marked the first demonstration of a role for a GPCR–C3BM signaling axis in cancer.

#### CXCR4

The chemokine receptor subfamily represents another group of GPCRs closely linked to cancer metastasis (68). Chemokine receptors respond to extracellular signaling molecules to regulate cellular processes such as chemotaxis, motility, and adhesion (69). One member of this subfamily, CXCR4, is overexpressed in a variety of malignancies and is well known to drive the metastatic spread of multiple cancers (70, 71). CXCR4 is stimulated by the ligand, stromal cell-derived factor 1α (SDF-1α; also referred to as CXCL12) (72). Interestingly, cells within the tumor microenvironment at common sites of metastasis, such as lung, liver, bone marrow, and lymph node often express high levels of SDF-1α. As a result, CXCR4 is thought to play a particularly important role in the homing of malignant cells to these metastatic sites and in promoting cell migration, growth, and survival at these distant sites (73, 74).

The fact that members of the chemokine receptor subfamily are known to promote activation of NF-κB *via* a PKC-dependent mechanism led investigators to hypothesize that a C3BM complex serves to mediate chemokine-induced NF-κB signaling. This hypothesis was first tested for CXCR4, and in 2009, CXCR4 became the second GPCR shown to utilize a C3BM signaling mechanism in cancer cells. Specifically, CXCR4 was found to activate NF-κB signaling through the C3BM complex in oral squamous cell carcinoma (OSCC) cells (Table 1A) (47). Stimulation of OSCCs with SDF-1α results in NF-κB activation that is suppressed by siRNA-mediated CARMA3, Bcl10, or MALT1 knockdown. Furthermore, knockdown of the C3BM components attenuates SDF-1α-induced OSCC invasion through a protein matrix. These important observations suggested that targeting the C3BM–NF-κB signaling pathway in OSCC may provide an important therapeutic opportunity for controlling metastasis of OSCC driven by SDF-1α. Moreover, since CXCR4-dependent signaling has been identified as a key regulator of cancer growth, invasion, and metastasis for a wide variety of other malignancies, including breast (75–77), ovarian (78), and prostate cancers (79–82), it will be of great interest to determine if the C3BM complex plays a key role in mediating SDF-1α dependent tumor progression or metastasis for these other tumor subtypes.

#### AGTR1

Our laboratory provided one of the first pieces of evidence for the existence of a C3BM signalosome by showing that each of the three components of this complex is indispensable for NF-κB activation induced by the hormone angiotensin II in hepatocytes (44). Since angiotensin II signaling is classically known for its role in promoting vascular inflammation and dysfunction, we initially focused our subsequent work in this area on implications for the AGTR1–C3BM signaling axis in vascular biology and atherogenesis (52). Yet, we also recognized that there was a growing body of literature implicating both angiotensin II and its receptor AGTR1 as key players in the pathogenesis of a variety of cancers, including squamous cell carcinoma of the skin (83), glioblastoma (84), gastric cancer (85), ovarian cancer (86), and others (87). Furthermore, in 2009, our group participated in a multidisciplinary bioinformatic meta-analysis effort which identified AGTR1 as the second most high-scoring gene expression outlier in breast cancer cases (88). As expected, HER2 (ERBB2) was the top-scoring outlier, with HER2 overexpression occurring in approximately 25–30% of cases. Yet, we noted significant AGTR1 overexpression in nearly as many cases (approximately 20%) and demonstrated that AGTR1 and HER2 overexpression are mutually exclusive in breast cancer. In addition, our subsequent bioinformatic analyses demonstrated that AGTR1 overexpression correlates with aggressive clinical features in breast cancer, including a higher rate of lymph node metastasis, reduced responsiveness to neoadjuvant therapy, and reduced overall survival (50).

Since HER2 and AGTR1 overexpression are mutually exclusive in breast cancer, we hypothesized that these two receptors likely activate redundant signaling pathways, critical for driving pathogenesis. Thus, because HER2-dependent NF-κB activation underwrites several of the hallmarks of cancer (89), we reasoned that AGTR1 might similarly drive breast cancer progression *via* an NF-κB-dependent mechanism, and possibly through the same C3BM signaling pathway that we had clearly described as operating in endothelial cells in the context of vascular pathobiology. Indeed, we found that that AGTR1 overexpression, in both breast cancer patient samples and breast cancer cell lines, drives NF-κB activation and an associated NF-κB gene expression signature; as we expected, CARMA3, Bcl10, and MALT1 are each required for this to occur (Table 1A) (50). Functionally, we found that activation of the AGTR1–C3BM–NF-κB signaling axis drives multiple breast cancer cell intrinsic responses including cell proliferation, migration, and invasion. In addition, we found evidence for cancer cell extrinsic effects in that the signaling axis drives expression and secretion of several cytokines and growth factors that can act on cells of the tumor microenvironment. These include IL-6, IL-8, Activin A, and IL-1β, among others, all of which influence endothelial cells, stromal cells, and immune cells. Indeed, we were able to demonstrate that conditioned media from AGTR1 + breast cancer cells drives endothelial chemotaxis through one or more of these secreted, C3BM-dependent factors. Furthermore, using a mouse model of AGTR1 + breast cancer, we found that suppression of C3BM signaling using an shRNA directed against Bcl10 results in significantly impaired tumor angiogenesis. Taken together, our study provided the first demonstration of a role for the C3BM complex in AGTR1 + breast cancer. Since several other GPCRs implicated as oncogenic drivers in breast cancer, including the LPARs, CXCR4, and PAR1, have been shown to stimulate NF-κB *via* a CARMA3-dependent pathway, albeit in other contexts, we speculate that the C3BM signalosome may act as a central signaling node for multiple subsets of breast cancer characterized by overexpression and/or hyperactivity of these related GPCRs.

### Other GPCRs Signal *via* the CARMA3-Containing CBM Complex

In addition to LPAR, CXCR4, and AGTR1 described above, several other GPCRs have been found to signal through the C3BM complex in cells outside of the immune system (Table 1B). In these specific cases (i.e., for ET_A_R, CXCR2, PAF-R, and PAR1), the GPCR–C3BM axis has so far been linked only to inflammatory processes and not to solid tumor pathogenesis. For example, the endothelin-1 receptor, ET_A_R, was one of the first GPCRs found to operate through the C3BM complex to activate NF-κB. Specifically, endothelin-1 treatment induces robust NF-κB activation in Bcl10^+/−^ MEFs but not in Bcl10^−/−^ MEFs (45). Intriguingly, there has been little additional work to explore the pathophysiologic consequences of C3BM signaling downstream of ET_A_R since this initial observation, and to our knowledge, there have been no studies to test its potential relevance to cancer biology. Nevertheless, several reports have recently emerged implicating endothelin-1/ET_A_R associated NF-κB activation in the pathogenesis of ovarian cancer (90), cervical cancer (91), prostate cancer (92), and chondrosarcoma (93). Thus, it is conceivable that the C3BM complex could play a pathogenic role downstream of ET_A_R in these cancers.

As another example, the interleukin-8 (IL-8/CXCL8) receptor, CXCR2, similar to CXCR4 described above, is a CXC-family GPCR cytokine receptor that operates upstream of the C3BM complex. IL-8 is critically involved in a wide array of cellular processes and has potent pro-inflammatory and proangiogenic signaling capacity. IL-8 stimulation of endothelial cells induces the expression of VEGF, and this occurs *via* a CXCR2–C3BM–NF-κB signaling pathway (46). The VEGF produced *via* this mechanism in turn acts in autocrine fashion on endothelial cells to further stimulate angiogenesis. The establishment of this feed-forward autocrine loop could therefore play a prominent role in tumor angiogenesis and cancer promotion, but the role for the IL-8-CXCR2–C3BM signaling axis has not yet been formally studied in the context of cancer biology.

PAF-R is another example of a GPCR that signals *via* the C3BM complex (48). The levels of platelet-activating factor (PAF), the ligand for PAF-R, are elevated in patients suffering from disorders associated with intestinal inflammation such as Crohn’s disease (94), ulcerative colitis (95), and neonatal necrotizing enterocolitis (96). It is thought that PAF causes intestinal injury *via* induction of NF-κB driven inflammation. In intestinal epithelial cells, PAF-R stimulation induces the interaction of Bcl10 with CARMA3, and Bcl10 deficiency abrogates PAF-R-dependent pro-inflammatory signaling (48). Currently, there is no known connection between PAF-R dependent C3BM signaling and the development of colon cancer associated with inflammatory bowel disease.

The thrombin receptor, PAR1, is one of the four members of the protease-activated receptor (PAR) family of GPCRs and is normally expressed in both platelets and endothelial cells (97, 98). In platelets, thrombin activation of PAR1 promotes platelet aggregation and coagulation, while in endothelial cells, PAR1 activation promotes an inflammatory response associated with enhanced endothelial permeability, immune cell attachment and adhesion, and the pathogenesis of diseases associated with endothelial dysfunction such as atherogenesis (99, 100). Our group found that the C3BM complex operates downstream of PAR1 in endothelial cells where it mediates thrombin-dependent NF-κB activation and expression of the cell-surface VCAM and ICAM adhesion molecules (49). As such, the C3BM complex controls thrombin-dependent adhesion of monocytes to the endothelial surface, which is an early step in the transmural migration of circulating monocytes into the vascular intimal space (49). In follow-up studies, we discovered that thrombin stimulation of endothelial cells induces MALT1 proteolytic activity and cleavage of the microtubule-binding protein, CYLD, resulting in microtubule destabilization (56). This destabilization then sets in motion a cascade of events that result in endothelial cell membrane retraction and increased vascular permeability (56). Together, these studies suggest that the C3BM complex plays a critical role in regulating both immune cell attachment to the endothelium and endothelial permeability to allow for transendothelial migration of immune cells. Thus, the C3BM complex may represent an important therapeutic target in the setting of vascular inflammatory disorders associated with endothelial dysfunction. As with the other GPCRs discussed in this section, the existing literature on the PAR1–C3BM signaling axis has not yet addressed any potential role in cancer biology.

Given the evidence of an important role for the CARMA3-containing CBM complex in mediating NF-κB signaling downstream of PAR1 and CXCR2 in endothelial cells, ET_A_R in fibroblasts, and PAF-R in intestinal epithelial cells, it will be of great interest to investigate the contribution of the CARMA3 complex to mediating the protumorigenic effects of each of these GPCRs. For example, PAR1 promotes the invasiveness of several types of cancer, including melanoma (101), breast cancer (102–104), and prostate cancer (105). Similarly, the IL-8/CXCR2 axis is thought to play a key role in promoting progression of a variety of tumors (106) including prostate cancer (107, 108), breast cancer (109), and melanoma (110). Since CXCR2 may be expressed on cancer cells, endothelial cells, and even immune cells, this signaling axis has the potential to exert protumorigenic effects both within the tumor cells themselves and also within cells of the tumor microenvironment to influence angiogenesis and the immune contexture. Thus, we speculate that there are still un-realized opportunities to establish novel links between CARMA3 and several other GPCRs in the pathobiology of selected solid tumors.

## Receptor Tyrosine Kinases (RTKs) also Signal *via* the CARMA3-Containing CBM Complex in Solid Tumors

Several growth factors, including platelet-derived growth factor (111), fibroblast growth factor (112), insulin-like growth factor (113), and epidermal growth factor (EGF) (114), induce NF-κB activation through receptors that are members of the RTK family. EGF and the EGF receptor EGFR (HER1) have been extensively studied in cancer, with EGFR amplification implicated in the pathogenesis of lung cancer (115), glioblastoma (116), and breast cancer (117), among others. Indeed, EGFR itself is the target of several therapeutics: either RTK inhibitors such as gefitinib, used in lung cancer, or monoclonal antibody inhibitors such as cetuximab, employed against colon cancer (118). Because EGFR is known to activate NF-κB through a PKC-dependent mechanism, the research group led by Xin Lin sought to investigate whether a CARMA3-containing CBM signaling complex might serve to bridge EGFR stimulation and NF-κB activation in cancer cells. In 2011, the Lin group demonstrated that EGF stimulation of breast cancer and epidermoid carcinoma cells leads to CARMA3-dependent NF-κB activation (Table 1C) (58). CARMA3 suppression in these cells was associated with an increase in cancer cell apoptosis, a loss of EGF-induced migration, and decreased tumor growth in a mouse xenograft tumor model. Excitingly, this study was the first demonstration of a receptor class outside of GPCRs capable of signaling through the CARMA3-containing CBM complex.

Following this initial discovery, the Lin group demonstrated that the C3BM complex is also required for activation of NF-κB by HER2, an EGFR-related RTK that is overexpressed in a large fraction of breast cancers (Table 1C) (59). They found that CARMA3 is required for the proliferation and survival of HER2 + breast cancer cells, and also required for HER2 to induce the upregulation of metalloproteinases that contribute to tumor metastasis. Moreover, they found that while overexpression of HER2 in the mouse mammary tissue leads to spontaneous development of mammary tumors, the growth of these tumors is significantly reduced in MALT1-deficient mice, providing evidence that the C3BM complex contributes to HER2-induced breast cancer progression *in vivo*. Thus far, EGFR and HER2 are the only RTKs that have been shown to signal *via* the C3BM complex. Future studies are needed to determine if other related RTKs might also utilize a CARMA3-dependent downstream signaling mechanism.

More recently, the Lin group used a functional genomics approach to identify a transmembrane protein of unknown function, TMEM43 (also referred as LUMA) as a new CARMA3-binding partner that serves as a critical mediator of EGFR-induced NF-κB activation in cancer cells (119). Interestingly, they found that TMEM43 is highly expressed in glioblastoma multiforme and that high TMEM43 expression correlates with poor survival outcome in patients with brain tumors. Furthermore, knocking out either CARMA3 or TMEM43 results in decreased growth and survival of human glioblastoma cells, both *in vitro* and *in vivo*. Based on these findings, the authors propose that TMEM43 serves to bridge EGFR and the C3BM complex. A role for TMEM43 in bridging the related RTK, HER2, to CARMA3 has not yet been investigated.

## Other Studies of CARMA3 in Solid Tumors

The role of the CARMA3-containing CBM complex in solid tumors has also been investigated outside the context of GPCR and RTK-driven NF-κB activation. Examples of some of these key studies of CARMA3 are reviewed below.

### Receptor-Independent Mechanisms for CARMA3–Bcl10–MALT1 Activation

One recent study examined the role of the C3BM complex in mediating DNA damage-induced NF-κB activation (120). While ionizing radiation and many chemotherapeutics used in the treatment of solid malignancies operate through induction of DNA damage and subsequent apoptosis, the molecular mechanism by which cancer cells upregulate DNA damage repair functions to resist these therapies has not been fully described (121). NF-κB has been known to play a pro-survival role in the context of irradiation or chemotherapeutic treatment, inducing cell cycle arrest and allowing for DNA damage repair and ultimately promoting therapeutic resistance and tumor survival. Given the involvement of the NF-κB transcription factor family in solid tumor DNA damage resistance (122), the laboratories of Xin Lin and Xuequiang Zhao asked whether the C3BM complex plays a role in this context (120). Intriguingly, they found that NF-κB induction by the chemotherapeutics etoposide or camptothecin require MALT1, suggesting a role for the C3BM complex in DNA damage-associated NF-κB activation. Furthermore, CARMA3, MALT1, and Bcl10 were each shown to be required for NF-κB induction by doxorubicin. The authors then exposed CARMA3^−/−^ mice to radiation and compared their survival to irradiated heterozygous CARMA^+/−^ littermate controls. Whereas all of the CARMA3^+/−^ mice survived 12 Gy radiation, CARMA3^−/−^ mice showed decreased radiation survival. Histological examination of tissue from irradiated mice showed decreased proliferation in CARMA3 homozygous knockouts, suggesting a role for the C3BM complex in post-radiation tissue repair. Finally, DNA damage-induced NF-κB activation appeared to be PKC-independent in cervical cancer cells, pointing to a novel upstream mechanism activating the C3BM complex and NF-κB signaling in response to DNA damage.

### Overexpression of CARMA3 in Cancer

The CBM complex generally acts to transmit intracellular signals from upstream receptors to downstream targets such as NF-κB in a controlled manner; however, enforced overexpression of CARMA3, Bcl10, or MALT1 in transfected cells can result in inappropriate CBM signaling and activation of downstream targets, independent of upstream input (5, 8, 35, 36). The precise mechanisms for this activation are not clearly understood, but may stem from C3BM oligomerization driven by concentration-dependent mass action. Recently, multiple groups have examined whether CARMA3 or other CBM proteins are overexpressed in cancer cells compared with normal tissue and whether CARMA3 overexpression correlates with increased cancer progression or lower rates of patient survival. In 2012, a semi-quantitative immunohistochemical (IHC) analysis of non-small-cell lung cancer specimens demonstrated that 70% of tumor samples exhibited increased CARMA3 staining relative to normal tissue from the same patients (57). Higher CARMA3 expression levels correlated significantly with advanced TNM staging and larger primary tumor size. The authors also reported a significant correlation between CARMA3 expression and expression of EGFR as well as EGFR mutation. In addition, they showed that CBM protein knockdown in lung cancer cell lines suppressed lung cancer cell proliferation and invasion *in vitro*.

CARMA3 expression studies have been performed in a variety of other solid tumors with similar results. In colon cancer, IHC analysis showed that approximately 31% of colon cancers harbor increased CARMA3 expression (123); CARMA3 knockdown in colon cancer cells resulted in decreased NF-κB activity and led to decreased proliferation and cell cycle arrest. In another report, 42% of breast cancer samples demonstrated increased CARMA3 expression by IHC, and CARMA3 expression level positively correlated with TNM staging. Furthermore, CARMA3 knockdown in breast cancer cells with high endogenous CARMA3 resulted in increased paclitaxel-induced apoptosis, while overexpression of CARMA3 resulted in enhanced proliferation and reduced apoptosis, results which suggested a role for CARMA3 in chemotherapeutic resistance (124). In glioma patient tumor specimens, 26% of samples showed CARMA3 overexpression relative to normal astrocytes in the same specimen (125); the authors also showed that CARMA3 knockdown in a glioma cell line suppresses cell invasion *in vitro* and results in decreased expression of MMP9, a matrix metalloprotease closely linked with tissue invasion. These results suggested a possible mechanism by which C3BM signaling promotes invasion in human glioma. Strong associations between CARMA3 expression level, tumor grade, and lymph node metastasis have also been reported in pancreatic and ovarian cancers, with 36 and 52% of samples showing increased CARMA3 expression, respectively (126, 127). In ovarian cancer, the authors also noted that CARMA3 knockdown increased the cytotoxic effects of cisplatin on tumor cells, again pointing to a possible mechanism by which C3BM signaling promotes resistance to genotoxic chemotherapy. Finally, a study of CARMA3 expression performed in renal cell carcinoma samples showed a significant association between CARMA3 expression and tumor size, stage, and metastases (128). Patients with renal cell carcinomas expressing high levels of CARMA3 had a significantly worse prognosis relative to patients with tumors of lower CARMA3 expression, indicating the possible utility of CARMA3 expression as a prognostic biomarker in renal cell carcinoma.

While the above studies provide evidence for an intriguing link between CARMA3 expression levels and the pathogenesis of several solid tumors, it remains unclear as to whether the naturally occurring CARMA3 overexpression observed in some cancer cells is sufficient to drive dysregulated C3BM signaling, as it is when CARMA3 is artificially overexpressed in transfected cells. Alternatively, it is possible that the elevated levels of CARMA3 seen in these cancers acts to simply sensitize cancer cells to signaling from upstream GPCRs or RTKs. Further work is required to integrate findings from CARMA3 overexpression studies in cancer with those from studies focused on overexpression of protumorigenic receptors.

### Regulation of CARMA3 by MicroRNAs (miRNAs)

While advances have been made in elucidating the role of C3BM signaling in solid tumors, the molecular mechanisms that regulate the expression of CARMA3, Bcl10, and MALT1 remain largely unknown. CARMA3 expression appears to be widely distributed in varied tissue types throughout the body, but the transcription factors and signaling networks responsible for the regulation of CARMA3 expression have not been fully investigated. Recently, two regulatory miRNAs, small single-strand noncoding RNA oligomers important in gene expression regulation through post-transcriptional mRNA silencing (129), have been reported to play a role in regulating CARMA3 levels. miR-195, previously described as a tumor suppressor in colorectal cancer, attenuates CARMA3 expression, and reduced expression of miR-195 has been associated with increased colorectal cancer metastasis to lymph nodes as well as poor prognosis (130, 131). Furthermore, the authors demonstrated that miR-195 expression suppresses colorectal cancer cell proliferation, MMP9 expression, and *in vitro* invasion. These effects of miR-195 could be reversed by CARMA3 overexpression, suggesting that miR-195-mediated downregulation of CARMA3 could be an important mechanism by which miR-195 acts as a colorectal cancer suppressor.

Recently, miR-24 was identified in bladder cancer as a second miRNA capable of targeting CARMA3 (132). miR-24 is downregulated in bladder cancer cells, supporting its role as a possible tumor suppressor (133). Furthermore, miR-24 overexpression blunts bladder cancer cell proliferation and invasion *in vitro* and appears to reduce expression of N-cadherin and vimentin, proteins important in cell adhesion and cytoskeletal structure, respectively, that are associated with the epithelial–mesenchymal transition (EMT). EMT is the process by which cancer cells undergo the substantial gene expression reprogramming necessary for the spread of cancer and formation of distant metastasis, and is reflected by a change in morphology and an increased capacity for tissue invasion (134). Overexpression of CARMA3 “rescued” bladder cancer cells with coexpressed miR-24, partially restoring proliferation and invasion *in vitro* and upregulating EMT markers.

Taken together, these two studies suggest that CARMA3 expression may be governed by miRNAs that act as tumor suppressors. Increased CARMA3 expression, in spite of these miRNAs, appears to promote tumor progression in specific cancers.

## Looking to the Future: Unanswered Questions and Conclusions

Among the myriad of unanswered questions surrounding the role of CARMA3 in solid tumor pathophysiology, four important and related issues remain unaddressed. First, are gain-of-function mutations in CARMA3, similar to those found in CARMA1 in B-cell lymphoma, present in any solid tumors? To date, no gain-of-function mutations of CARMA3 have been reported in cancer or in any other disease. Second, what are the precise molecular mechanisms by which upstream GPCRs and/or RTKs regulate C3BM activity? Compared to antigen receptor engagement of CARMA1, much less is known about the intermediate proteins linking either GPCRs or RTKs to CARMA3. Is CARMA3, like CARMA1, subject to receptor-dependent phosphorylation and subsequent conformational change, thus allowing interaction with Bcl10, formation of a polymeric CBM complex, and subsequent downstream signaling? While inhibitor studies have implicated a role for PKCs in GPCR–C3BM signaling (43, 51, 53, 135), definitive phosphorylation of CARMA3 by PKCs, or any other kinase, has not yet been demonstrated. In addition, biophysical studies examining the detailed structural properties of the CARMA3-containing CBM complex, like those describing the CARMA1-containing complex (18, 19, 21), have not yet been reported.

Third, what is the role of MALT1 proteolytic activity, which is required for the survival of lymphoma cells harboring B-cell receptor or CARMA1 gain-of-function mutations, in tumors characterized by GPCR or RTK-driven CARMA3 activation? Our laboratory recently found that that stimulating the PAR1 receptor induces MALT1 protease activity in endothelial cells, providing the first evidence that a GPCR–CARMA3 signaling axis can trigger MALT1 proteolytic activity (56). It is important to point out that these studies were performed in endothelial cells and that MALT1 protease activity has not yet been evaluated in GPCR or RTK-driven CARMA3-dependent solid tumors. Based on our findings in endothelial cells, it seems likely that MALT1 proteolytic activity will be induced by oncogenic cell-surface receptors such as PAR1, AGTR1, CXCRs, and LPARs and will play a role in mediating the pro-tumorgenic effects of these receptors in solid tumors.

Fourth, what are the downstream molecular mechanisms by which CARMA3-dependent CBM signaling promotes tumorigenesis? Most of the existing literature has focused on NF-κB-dependent transcriptional reprogramming as the critical downstream event in GPCR/RTK-C3BM-driven tumorgenesis. Since the C1BM complex in lymphocytes is known to activate other transcription factors, such as Jun N-terminal kinase (JNK), in addition to NF-κB (136, 137), it seems possible that the C3BM complex in solid tumor cells may also activate JNK-dependent transcription, as well as other transcription factors. In addition, it will be important to consider potential transcription-independent mechanisms by which C3BM signaling could promote tumorgenesis. For example, several studies in immune cells have suggested that Bcl10/MALT1 can regulate actin and membrane remodeling *via* a transcription-independent mechanism (138, 139). It is thus possible that Bcl10/MALT1 may promote tumorigenesis in GPCR/RTK-C3BM-driven solid tumors *via* mechanisms that do not involve transcriptional reprogramming.

The answers to these questions will help to determine whether targeting specific receptors that drive CARMA3 signaling, targeting the C3BM signaling complex itself, or targeting the MALT1 protease, could represent promising therapeutic approaches for treating specific solid tumors. It will also be of interest to consider how the C3BM complex in solid tumors might be specifically targeted without impacting the C1BM complex in lymphocytes. Perhaps future structural analyses of the C3BM complex will reveal critical differences between how CARMA3 and CARMA1 interact with Bcl10/MALT1 that could be harnessed in designing novel protein–protein inhibitors specific to the C3BM? Alternatively, it may be possible in the future to specifically inhibit C3BM by engineering tissue/cell type-specific delivery of MALT1 protease inhibitors.

The current body of knowledge of CARMA3 and C3BM signaling in solid tumors paints a picture of an intracellular signaling pathway associated with numerous steps in solid tumor pathophysiology: from tumor growth to chemotherapeutic resistance, EMT, angiogenesis, invasion, and metastasis. Furthermore, CARMA3-dependent signaling has been implicated in the pathogenesis of a striking variety of solid tumors, suggesting that targeting the C3BM pathway could have wide-ranging therapeutic application in clinical oncology. With the body of research focused on CARMA3 in solid tumors built over this last decade, we now await the next decade of C3BM research with great anticipation and hope for novel treatments for our patients.

## Author Contributions

JM wrote the initial draft of the manuscript text. TF and PE prepared figures and tables and reviewed/edited the text. PL and LM-L supervised all aspects of manuscript preparation and performed final editing.

## Conflict of Interest Statement

The authors declare that the research was conducted in the absence of any commercial or financial relationships that could be construed as a potential conflict of interest.
